# Genetic association of *APOA5* and *APOE* with metabolic syndrome and their interaction with health-related behavior in Korean men

**DOI:** 10.1186/s12944-015-0111-5

**Published:** 2015-09-13

**Authors:** Ki Young Son, Ho-Young Son, Jeesoo Chae, Jinha Hwang, SeSong Jang, Jae Moon Yun, BeLong Cho, Jin Ho Park, Jong-Il Kim

**Affiliations:** Department of Family Medicine, Seoul National University College of Medicine, Seoul, Republic of Korea; Department of Biochemistry and Molecular Biology, Seoul National University College of Medicine, Seoul, Republic of Korea; Department of Biomedical Sciences, Seoul National University Graduate School, Seoul, Republic of Korea; Genomic Medicine Institute, Medical Research Center, Seoul National University, Seoul, Republic of Korea; Cancer Research Institute, Seoul National University College of Medicine, Seoul, Republic of Korea; Health Promotion Center, Seoul National University Hospital, Seoul, Republic of Korea

**Keywords:** *APOA5*, *APOE*, Health behavior, Metabolic syndrome, Triglyceride

## Abstract

**Background:**

Genome-wide association studies have been used extensively to identify genetic variants linked to metabolic syndrome (MetS), but most of them have been conducted in non-Asian populations. This study aimed to evaluate the association between MetS and previously studied single nucleotide polymorphisms (SNPs), and their interaction with health-related behavior in Korean men.

**Methods:**

Seventeen SNPs were genotyped and their association with MetS and its components was tested in 1193 men who enrolled in the study at Seoul National University Hospital.

**Results:**

We found that rs662799 near *APOA5* and rs769450 in *APOE* had significant association with MetS and its components. The SNP rs662799 was associated with increased risk of MetS, elevated triglyceride (TG) and low levels of high-density lipoprotein, while rs769450 was associated with a decreased risk of TG. The SNPs showed interactions between alcohol drinking and physical activity, and TG levels in Korean men.

**Conclusions:**

We have identified the genetic association and environmental interaction for MetS in Korean men. These results suggest that a strategy of prevention and treatment should be tailored to personal genotype and the population.

**Electronic supplementary material:**

The online version of this article (doi:10.1186/s12944-015-0111-5) contains supplementary material, which is available to authorized users.

## Background

According to a World Health Organization report, cardiovascular diseases were the cause of 30 % of all deaths globally in 2008 and the number of deaths is constantly increasing [1]. Metabolic syndrome (MetS) is a cluster of health conditions, which are strongly associated with the incidence of cardiovascular diseases and mortality [2–4]. The age-adjusted prevalence of MetS was 31.3 % [5], and a population-attributable fraction of MetS for cardiovascular disease was 12–17 % in Korean [6]. Thus, MetS is one of the most important current public health problems in Korea.

MetS is caused by multiple genetic and environmental risk factors, and an interaction between the factors has been widely postulated. Among the environmental factors, lifestyle factors such as a caloric excess diet and a sedentary lifestyle are important risk factors for MetS [7]. However, there is also evidence of the role of heredity for risk of MetS. Large proportion of variance of MetS components were associated with narrow-sense heritability explained by common SNPs (46 % for waist hip ratio, 30 % for glucose metabolism, 34 % for triglyceride, 25 % for HDL and 80 % for SBP) [8].

Genome-wide association studies (GWASs) have been used extensively to identify common genetic variants linked to MetS. Most of these variants were located in the genes involved in lipid metabolism (*PPARG* rs1801281, *APOA5* rs662799, *APOC3* rs2854117, *CETP* rs708272, *GNB3* rs5433 and *APOE* rs7412) [9]. Some of these single nucleotide polymorphisms (SNPs) were also associated with weight regulation, glucose metabolism and blood pressure. In addition, *FTO* rs9939609 was associated with weight regulation, and *TCF7L2* rs7903146 was involved in glucose metabolism [9].

However, most previous studies were conducted mainly in western populations. Few studies have been conducted in Asian populations of the association between MetS and its components, and specific loci [9, 10]. To our knowledge, no study has been conducted to evaluate the association between various genetic variants and MetS in Koreans. Furthermore, the interplay between health-related behaviors as environmental factors and genetic factors has not been fully evaluated in previous association studies. Thus, this study aimed to evaluate the association between previously studied SNPs and MetS, and the interaction between health-related behaviors and SNPs in Korean men. We hypothesized that genetic variants which were known to be related to MetS or its components in other population would be associated with MetS in Koreans, and this association would be modified by health-related behaviors.

## Results

### Population characteristics

Descriptive characteristics of the 254 participants with MetS and 939 control participants included in this study are shown in Table. 1. As expected, participants with MetS showed significantly increased risk levels for all of the MetS component variables (Waist circumference, high density lipoprotein, triglyceride, fasting glucose, systolic blood pressure and diastolic blood pressure) and BMI (*P* < 0.001). Of the health-related behaviors, participants with MetS showed higher exposure to smoking and drinking than controls, but their patterns of physical activity were similar.

**Table 1:** Characteristics of study subjects

Characteristics	Case with MetS	Control	*P*-value
	Mean ± SD or number (%)	Mean ± SD or number (%)	
Total number	254	939	
Age (years)	47.55 ± 6.56	47.92 ± 6.99	0.454
BMI (kg/m^2^)	26.43 ± 2.49	23.72 ± 2.43	<0.001
MetS components			
WC (cm)	94. 23 ± 6.38	85.92 ± 6.88	<0.001
HDL cholesterol (mg/dL)	44.09 ± 11.60	52.29 ± 11.49	<0.001
Triglyceride (mg/dL)	209. 16 ± 90.55	107.31 ± 57.42	<0.001
Fasting glucose (mg/dL)	105.87 ± 22.08	90.60 ± 12.99	<0.001
SBP (mmHg)	131.22 ± 13.08	120.95 ± 13.55	<0.001
DBP (mmHg)	83.90 ± 10.38	76.50 ± 9.85	<0.001
Health-related behaviors			
Smoking			0.017
Non smoker	46 (18.1 %)	217 (23.1 %)	
Former smoker	92 (36.2 %)	367 (39.1 %)	
Current smoker	116 (45.7 %)	355 (37.8 %)	
Drinking			0.002
Non drinker	39 (15.4 %)	176 (18.7 %)	
Light drinker	85 (33.5 %)	373 (39.7 %)	
Moderate drinker	40 (15.7 %)	157 (16.7 %)	
Heavy drinker	90 (35.4 %)	233 (24.8 %)	
Physical activity category			0.295
Low level	109 (44.9 %)	379 (41.3 %)	
Moderate level	87 (35.8 %)	341 (37.1 %)	
High level	47 (19.3 %)	198 (21.6 %)	

*P*-value represents the significance of the difference between case with MetS and control. All continuous variables were compared using *t* test. Health-related behaviors were assessed according to the linear-by-linear association of chi-square test

*BMI* body mass index, *MetS* Metabolic syndrome, *WC* waist circumference, *HDL* high density lipoprotein, *SBP* systolic blood pressure, *DBP* diastolic blood pressure, *SD* standard deviation

### Genetic associations

A list of genotyped SNPs is provided in Additional file 1: Table S1. The association of 17 SNPs with MetS and its components was tested using logistic regression analysis. After a basic adjustment (model 1), one SNP (rs662799) near *APOA5* showed the most significant association with increased risk of elevated triglyceride (TG) levels (*P* = 3.25 × 10^−6^), low levels of high-density lipoprotein (HDL) (*P* = 7.20 × 10^−4^), and MetS (*P* = 2.90 × 10^−4^). Another SNP (rs769450) in *APOE* showed significant association (*P* = 0.0015) with decreased levels of TG (Table 2, and Additional file 1: Tables S2 and S3).

**Table 2:** The logistic regression results of significant SNPs for MetS and its components

Gene	SNP	Chr	Model	Phenotype (number of case / control)											
				WC (460 / 733)		TG (348 / 845)		HDL (198 / 995)		BP (451 / 742)		Glucose (304 / 889)		MetS (254 / 939)	
				OR (95%CI)	*P*-value	OR (95%CI)	*P*-value	OR (95%CI)	*P*-value	OR (95%CI)	*P*-value	OR (95%CI)	*P*-value	OR (95%CI)	*P*-value
*APOA5*	rs662799	11	Model 1^a^	0.971 (0.80–1.17)	0.7550	**1.609** (1.32–1.97)	**3.25E-06**	**1.510** (1.19–1.92)	**7.20E-04**	1.033 (0.85–1.25)	0.7379	1.042 (0.84–1.29)	0.7019	**1.498** (1.20–1.86)	**2.90E-04**
			Model 2^b^	1.009 (0.83–1.22)	0.9247	**1.726** (1.40–2.13)	**2.65E-07**	**1.654** (1.29–2.12)	**7.04E-05**	1.016 (0.84–1.23)	0.8766	1.100 (0.88–1.37)	0.3927	**1.603** (1.28–2.01)	**3.96E-05**
*APOE*	rs769450	19	Model 1^a^	0.894 (0.72–1.11)	0.3091	**0.674** (0.53–0.86)	**0.0015**	1.072 (0.81–1.41)	0.6235	0.869 (0.70–1.08)	0.2080	0.994 (0.78–1.26)	0.9630	0.759 (0.58–0.99)	0.0427
			Model 2^b^	0.893 (0.72–1.11)	0.3130	**0.653** (0.51–0.84)	**7.72E-04**	1.016 (0.76–1.36)	0.9152	0.896 (0.72–1.12)	0.3316	0.979 (0.77–1.25)	0.8714	0.757 (0.58–0.99)	0.0448

MetS components cut-off: WC >90 Cm for men, TG > 150 mg/dL, HDL < 40 mg/dL, BP: SBP > 130 mmHg or DBP > 85 mmHg, Glucose > 100 mg/dL

Bold indicates significance of Bonfferoni adjusted *P*-value (<0.0029)

*WC* waist circumference, *TG* triglyceride, *HDL* high density lipoprotein, *BP* blood pressure, *MetS* Metabolic syndrome

^a^Model 1: Adjusted for age and region of recruitment

^b^Model 2: Further adjusted for smoking, alcohol and physical activity

After model 1 adjustments, linear regression analysis revealed that rs662799 of *APOA5* was significantly associated with elevated TG levels (*P* = 1.27 × 10^−6^) and low levels of HDL (*P* = 4.22 × 10^−4^) (Additional file 1: Table S4). The rs769450 polymorphism of *APOE* was found to be moderately associated with decreased TG levels, but the association was not significant after Bonferroni adjustment.

**Fig. 1:**
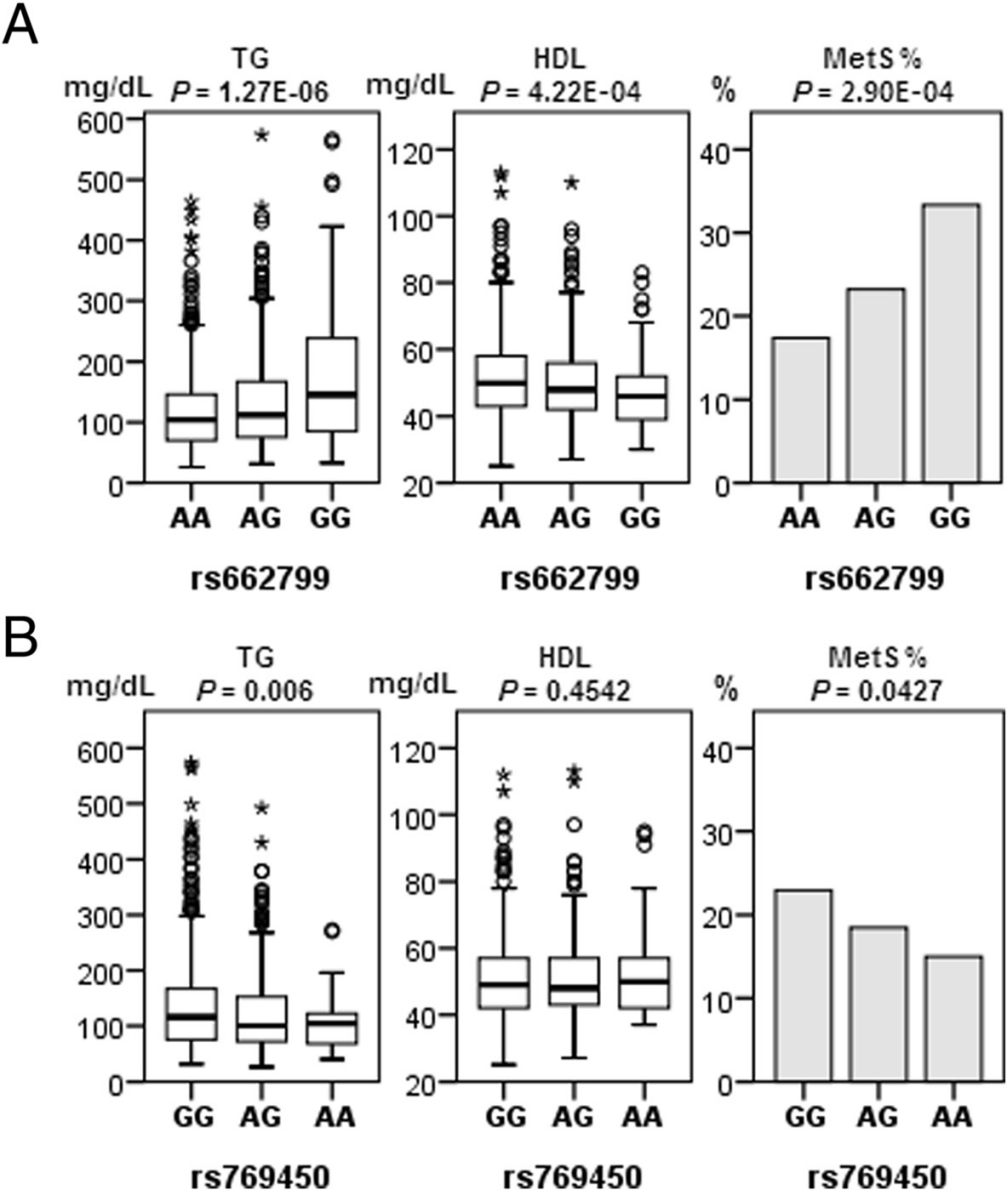
The additive effect of rs662799 and rs769450. TG, HDL level and MetS prevalence according to the (**a**) rs662799 and (**b**) rs769450 genotypes. The *P*-values of TG and HDL were estimated via linear regression analysis and MetS prevalence was estimated via logistic regression analysis. All analyses were adjusted for age and region of recruitment (Model1). HDL: high density lipoprotein, TG: triglyceride

The significantly associated TG and HDL levels, and MetS prevalence are shown in Fig. 1, according to the genotype of rs662799 and rs769450. A significantly additive effect of rs662799 is shown for TG and HDL levels, and MetS prevalence, because it increased the risk of MetS. A moderate additive effect is shown for rs7694450 for decreased TG levels and MetS prevalence.

### Health-related behavior and genotype-stratified analysis

**Fig. 2:**
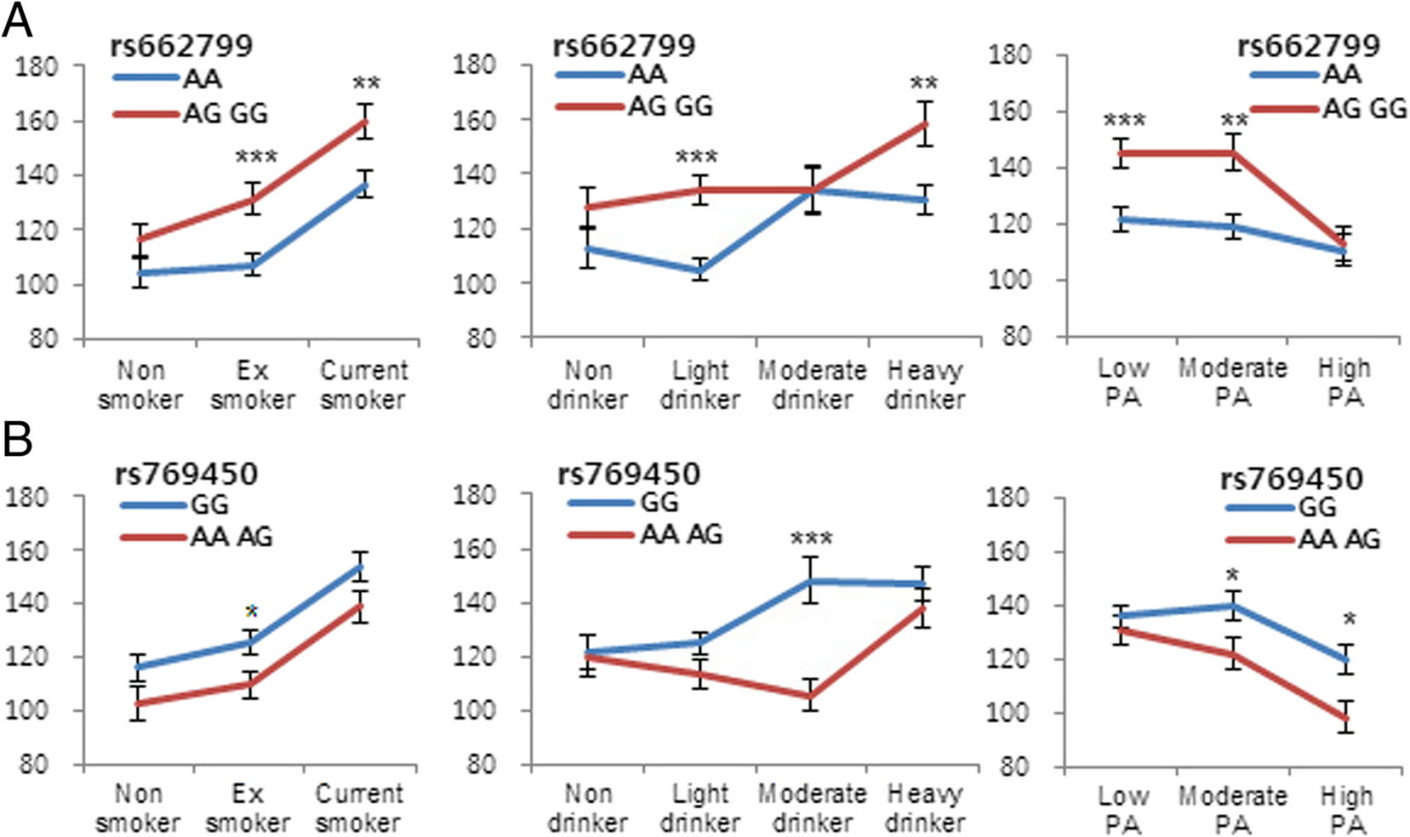
Significant SNPs and their interaction with health-related behaviors. TG levels in participants with (**a**) rs662799 and (**b**) rs769450 genotypes associated with stratified levels of smoking, drinking and physical activity. Asterisks indicate the significance of the differences in TG levels between each genotype (*t-*test, *P < 0.05, **P < 0.01, and ***P < 0.001). All error bars represent standard errors. Stratified drinking cutoff levels, alcohol (g)/week: nondrinker = 0; light drinker <98 g; moderate drinker >98 g but <196 g; and heavy drinker ≥196 g. PA: physical activity, TG: triglyceride

We conducted a multiple regression analysis with two adjustment models (model 1: age and region of recruitment; model 2: age, region of recruitment, smoking, alcohol and physical activity). We obtained a more significant result after health-related behaviors were included in the adjustment (model2) (Table 2). All health-related behaviors were significantly associated with TG levels (data not shown). We analyzed the relationship between the two SNPs significantly associated with TG levels within stratified levels of health-related behaviors (Fig. 2, Additional file 1: Figure S1). We compared TG levels between major homozygous genotype vs the sum of heterozygous and minor homozygous genotypes, because of the lower number of minor homozygous genotypes.

First, participants were stratified according to their smoking status. Regarding rs662799, the group with the risk allele (AG and GG genotypes) showed higher levels of TG than the reference (AA genotype) group did at all levels of smoking, especially for ex-smokers (*P* = 0.0004) and current smokers (*P* = 0.0041) (Fig. 2a). However, we could not find any interaction between smoking and the rs662799 genotype (Additional file 1: Figure S1A). Regarding rs769450, the AA and AG genotypes showed a protective effect by decreasing the TG levels compare with the reference (GG genotype) for all smoking exposure; however, only ex-smokers showed significantly different levels of TG (*P* = 0.023), and a specific interaction between smoking and rs769450 was not found (Additional file 1: Figure S1B).

Second, participants were stratified by level of exposure to alcohol. Regarding rs662799, AG and GG genotype showed higher TG levels compare with the reference (AA genotype), except in moderate drinkers. The reference group (AA genotype) exhibited lower TG levels in non- and light drinker, whereas TG levels were dramatically increased in moderate drinkers. The group with risk allele (AG and GG genotype) showed similar TG levels in non-, light and moderated drinkers, whereas TG levels were increased in heavy drinkers. Regarding rs769450, TG levels were decreased in non-, light and moderate drinkers in the group with the AA and AG genotype, in contrast to participants in the group with the reference (GG genotype). Consequentially, in moderate drinkers, significantly different (*P* = 4.72 × 10^−5^) levels of TG were found according to the rs769450 variant.

Third, we evaluated the differences in TG levels according to genotype and stratified physical activity. Regarding rs662799, participants in the group that carried the AG and GG genotypes had significantly higher TG levels compared with those with reference (AA genotype) in the low (*P* = 0.0006) and moderate (*P* = 0.0011) physical activity groups. However, individuals with the AG and GG genotypes and high levels of physical activity showed dramatically decreased levels of TG. Regarding rs769450, participants with low physical activity in the group that carried the AA and AG genotypes showed similar TG levels compare with those of reference (GG genotype). However, a more pronounced decrease in the levels of TG were found in the group with the AA and AG genotypes in cases with increased levels of physical activity (*P* = 0.027 and 0.013, moderate and high physical activity, respectively).

## Discussion

We have demonstrated that SNPs in *APOA5* and *APOE* were significantly associated with MetS and its components in the Korean men who participated in this study. We also evaluated the interaction between these SNPs and health-related behaviors affecting the levels of TG.

Generally, SNPs of *APOA5* are associated with dyslipidemia, which is a component of MetS [9, 11]. Four common SNPs (rs662799, rs2266788, rs3135506 and rs2072560) verified in the *APOA5* gene, are associated with increased TG levels [12]. Among these SNPs, rs662799 is located in the promotor region of *APOA5* gene, which is strongly associated with elevated TG levels, and confers risk for MetS and stroke [13, 14]. In Koreans, there is an association between rs662799, a risk of MetS and increased susceptibility to diabetes mellitus [15, 16].

In this study, we found that rs662799, which is located near *APOA5*, was significantly associated with levels of TG, HDL and the prevalence of MetS in Korean men (Table 2). The minor allele frequency (MAF) of rs662799 was 0.293, which is similar to that in Koreans (0.302) and Chinese (0.284 and 0.304), but higher than the frequency reported in Europeans (0.095) [15, 17, 18]. A study of Koreans [15] showed that, among the MetS components, TG and HDL were equally significantly associated with rs662799, with a weak association (*P* = 0.015; odds ratio [OR], 1.285) between MetS and rs662799 in men. However, we found more significant association (*P* = 2.9 × 10^−4^; OR, 1.498; model 1) between MetS and rs662799 in men (Table 2).

The *APOA5* 3′-UTR variant, rs2266788, is associated with plasma TG levels through downregulation of *APOA5* [19]. In the present study, the *APOA5* SNP, rs2266788, showed marginal association with TG and MetS. After multiple testing correction, this SNP was found to be significantly associated with TG, but only in adjustment model2 (Additional file 1: Tables S2 and S3).

*APOE* functions in the catabolism of chylomicron remnants and very low-density lipoprotein from plasma. Two common SNPs located in *APOE* exon 4 (rs429358 and rs7412) encode three *APOE* isoforms (E2, E3 and E4). These isoforms have been studied extensively and found to contribute to MetS, cardiovascular disease, hypertriglyceridemia and ischemic stroke [20–24]. However, other common variants have not yet been sufficiently explored. The common *APOE* intron SNP, rs769450, was associated with low-density lipoprotein in Europeans-Americans and African-Americans [25]. To our knowledge, the association between rs769450 and MetS or TG levels has not been reported.

In the present study, we found that rs769450 of *APOE* is significantly associated with TG levels. A protective effect was shown for rs769450, not only for TG levels, but also moderately for MetS (Fig. 1b). The MAF of rs769450 was 0.202 and lower than the frequency in Europeans (0.437) [26]. A number of studies of *APOE* have focused on two common SNPs (rs429358 and rs7412) on exon 4, which make three *APOE* isoforms. These three *APOE* isoforms show a significant association with TG levels and MetS in various populations. In a Japanese study of 1788 participants, rs7412 was found to be associated with MetS [27]. However, we could not find any significant association between rs7412 and MetS (*P* = 0.6362) or TG level (*P* = 0.2782).

Although it seemed that there was no interaction between cigarette smoking and *APOA5* rs662799 and *APOE* rs769450, we found interactions between the genotypes and alcohol drinking and physical activity that affected TG levels. The difference between TG levels was narrowed for participants with *APOA5* rs662799, but widened for *APOE* rs769450 in moderate drinkers, and participants with high levels of physical activity.

Regarding rs662799, participants in the group with the AG and GG genotypes had similar levels of TG whether they were non-, light, or moderate drinkers. However, moderate drinkers in the group with the AA genotype showed significantly increased levels of TG (Additional file 1: Figure S1A). The difference in TG level between participants with different SNPs was insignificant for moderate drinkers. We observed that the levels of TG were dramatically decreased in participants in the group with the AG and GG genotypes of rs662799 and high levels of physical activity.

Regarding rs769450, the AA and AG genotypes had a protective effect in non-, light and moderate drinkers, who showed marginally decreased TG levels. High levels of physical activity had more benefit in decreasing TG levels in participants with the AA and AG genotypes than in those without them. This finding regarding physical activity is consistent with clinical guidelines, which recommend physical activity to reduce cardiovascular diseases, and indicate that high levels of physical activity should be emphasized in general. We found that variants of rs769450 had more benefit in decreasing TG levels in those participants who drank moderately and had high levels of physical activity, than in those without this variant.

Although moderate drinking and high physical activity could be recommended in general, we can assume that these behaviors are of greater benefit in people with specific genotypes. In this study, participants with variant SNPs of *APOA5* and *APOE* showed a greater decrease in TG levels when they exhibited these behaviors. Therefore, tailored consultation for health-related behaviors should be considered if genetic information is available in clinical situations.

Together, our findings show that rs667799 and rs769450 are significantly associated with elevated or decreased levels of TG, respectively and MetS in Korean men. In Koreans, the MAF of the risk SNP (rs662799) is higher, and the MAF of the protective SNP (rs769450) is lower than in Europeans. Consequently, East Asians, including Koreans, are more exposed to the risk of MetS than Europeans. Thus, in Korean men, a tailored approach that addresses environmental factors is important in order to overcome the unfavorable genetic predisposition.

Despite variants of *APOA5* and *APOE* revealing an increase in the risk of MetS in Koreans, other SNPs that are associated with MetS in other populations were not shown to be significantly associated with MetS or its components in Koreans after multiple test correction. These findings suggest that susceptibility to MetS is different in various populations according to their genotypes. Therefore, effective strategies for the prevention and treatment of MetS may differ according to the genotype and environmental factors in each population.

A limitation of this study was that we only tested the associations in men. According to the study design, we could not evaluate the same association in the general population or only in women. Further studies with more participants, including women, are warranted to validate our results. Recall bias should also be considered, because data on health-related behavior were collected via a self-administered questionnaire. In addition, we could not infer temporary relationship because this study was cross-sectional study, so that causality could not be confirmed. And other potential confounders related to MetS such as nutritional information including nutriceuticals, were not available, we could not include such variables in analysis.

## Conclusions

In conclusion, rs662799 of *APOA5* and rs769450 of *APOE* were significantly associated with regulated TG levels and MetS in the Korean men we studied. These SNPs appeared to interact with alcohol drinking and physical activity, thereby affecting TG levels. Because East Asians, including Koreans, more frequently have these risk SNPs than Europeans, strategies for the prevention of MetS and its treatment should be tailored to personal genotype and health-related behaviors.

## Methods

### Study design and population

We conducted a cross-sectional study designed to evaluate the association between genetic variants and biological markers such as anthropometric measurement, visceral fat, MetS components. The participants in this study were general males who visited the Health Promotion Center and Healthcare System Gangnam Center at Seoul National University Hospital from December 2009 to December 2013.

Patients visited the center to undergo a periodic health checkup and every patient met a physician before the health checkup. They completed a self-administered questionnaire, which included questions on health-related behaviors. Participants were informed about the study before their health checkup, and asked to participate. Written informed consent was obtained by their physician.

Every single male patient under 60 years who visited the centers for regular health checkup and received abdominal computed tomography in the period was asked to participate in this study. Patients were excluded from study when they did not agree to participate in this study, had thyroid functional diseases, took medication or treatment regarding weight control, diabetes mellitus or psychiatric diseases, experienced weight change over 10 % of their weight for last 3 months, or had comorbidity such as diabetes mellitus, coronary heart diseases, cerebrovascular diseases and cancers except skin cancers.

Totally 1563 patients agreed to participate in this study and we obtained informed consent from these patients. Among them, 54 patients were excluded from analysis. Twenty six patients were excluded because it was found that they were over 60 years, 20 patients were excluded because of medication history or comorbidities, 7 patients were excluded because of duplication. In addition, we excluded 317 patients who took medication for hypertension or hypercholesterolemia. Finally 1193 patients were included in analysis. Anthropometric measurements and laboratory tests were conducted as part of a general health checkup, and genomic DNA was extracted from peripheral blood leukocytes of all participants using the QuickGene DNA whole-blood kit with QuickGene-610 L equipment (Fujifilm, Tokyo, Japan), according to standard protocols. The study protocol was approved by the Institutional Review Board (approval number, H-0911-010-299) of the Seoul National University Hospital, Korea.

### Phenotype measurement

Eligible participants completed a questionnaire designed for clinical purposes, which included the following questions: (a) demographic details (sex, age, household income, education and marital status); (b) comorbidities and medication for conditions including hypertension, diabetes mellitus and hypercholesterolemia; and (c) health-related behavior (cigarette smoking, alcohol drinking and physical activity).

Body weight and height were measured with light clothing and no shoes. Body mass index (BMI) was calculated as body weight (in kg) divided by the square of height (in m^2^). Waist circumference was measured from the narrowest point between the lowest point of the rib and the highest point of the iliac crest. Blood pressure (BP) was measured in a sitting position at least 5-min rest period. The second and third systolic and diastolic blood pressure were averaged for this analysis. Blood sample was taken in the morning with at least 8 h of fasting state. All blood samples were taken on the same day.

A consensus definition of MetS for clinical diagnosis was proposed by International Diabetes Federation Task Force in 2009 [28], and we used this definition. According to the definition, MetS could be diagnosed if patients had three or more of the following five MetS features: increased waist circumference, in which we used the Korean obesity criterion (≥90 cm) in place of the original criterion (≥94 cm) [29]; low levels of HDL cholesterol (<40 mg/dL); elevated levels of TG (≥150 mg/dL); elevated blood pressure (≥130/85 mmHg); and elevated glucose levels (≥100 mg/dL). We excluded patients who took medication for hypertension, hypercholesterolemia, or diabetes mellitus.

For health-related behavior, current cigarette smoking, problem alcohol drinking and physical inactivity were defined as risky health-related behaviors. According to National Health Interview Survey, participants who had smoked more than 100 cigarettes in their lifetime, and smoked every day or on some days were defined as current smokers [30]. Those who had smoked more than 100 cigarettes and stopped smoking were defined as ex-smokers. In line with definition of National Institute on Alcohol Abuse and Alcoholism, we defined moderate alcohol consumption as up to 2 drinks per day, where a drink was 14 g of alcohol [31]. In addition we defined light drinkers as less than 1 drink per day to examine dose—response relation. Therefore men who drank ≥196 g of alcohol per week were defined as heavy drinkers; men who drank ≥98 g but <196 g of alcohol were defined as moderate drinkers; and men who drank <98 g were defined as light drinkers. Physical activity was measured as daily physical activity using the Korean version of the International Physical Activity Questionnaire-Short Form (IPAQ-SF), which was translated into Korean and validated [32]. Physical activity was categorized as low, moderate, or high using the IPAQ scoring protocol [33].

### SNP selection and genotyping

A total of 17 SNPs with a MAF of less than 0.05 in the Asian HapMap (CHB + JPT) were selected to test associations with MetS and its components (Additional file 1: Table S1). Among selected SNPs, *PPARG* rs1801281, *APOA5* rs662799, rs2266788 *CETP* rs708272, rs1800775 *GNB3* rs5433, *APOE* rs769450, rs7412 *FABP2* rs1799883 and *ENPP1* rs1044498 are involved in lipid metabolism [9, 19, 25, 34–36]. Some of these SNPs are also involved in weight regulation, glucose metabolism and blood pressure regulation. In addition, *LMNA* rs4641 is involved in insulin resistance and glucose metabolism [37]. *LEPR* rs1137101 is associated with circulatory leptin level, while *ADIPOQ* rs1501299, rs2241766 and rs266729 are associated with circulatory adiponectin [38]. *ADRB2* rs1042714 and *ADRB3* rs4994 are in genes that encode the β-adrenoreceptor and mediate the action of catecholamines, respectively [39, 40]. All SNPs were genotyped using TaqMan assays and a ViiA7 genotyping system (Applied Biosystems, Foster City, CA). To maintain the quality of the genotyping data, a genotyping call rate > 95 % and Hardy-Weinberg equilibrium *P* > 0.01 were considered.

### Statistics

We conducted the genetic association analysis between the chosen SNPs and MetS and its components via an additive model using PLINK software, version 1.07 [41]. Logistic regression analysis was used to test the association between each SNP and MetS risk categorized groups, adjusted using two different methods (model 1: age and region of recruitment; model 2: age, region of recruitment, smoking, alcohol and physical activity). A linear regression analysis was conducted to investigate the association between chosen SNPs and quantitative MetS risk phenotypes in the whole cohort, with adjustment for the same models. A Bonferroni-adjusted *P* < 0.0029 (0.05/17 SNPs) was considered significant for the multiple test. For interaction analysis, TG level was compared between each genotype among same stratified health-related behaviors (Fig. 2) and between each stratified health-related behaviors among same genotype (Additional file 1: Figure S1) using a *t-*test. Demographic data arrangement and interaction with health-related behaviors were analyzed using IBM SPSS Statistics for Windows, version 20 (IBM Corp., Armonk, NY, USA).

## Additional file

Additional file 1: Table S1. The list of selected SNPs. **Table S2.** The logistic regression results for MetS and its components (Model 1). **Table S3.** The logistic regression results for MetS and its components (Model 2). **Table S4.** The linear regression results of significant SNPs for quantitative trait of MetS components. **Figure S1.** TG level difference between health-related behaviors. (DOCX 75 kb)

## Abbreviations

BP: Blood pressure
HDL: High density lipoprotein
GWAS: Genome wide association study
MetS: Metabolic syndrome
MAF: Minor allele frequency
SNP: Single nucleotide polymorphism
TG: Triglyceride
