# Supplementary material for: Genetic association of APOA5 and APOE with metabolic syndrome and their interaction with health-related behavior in Korean men
Source: Lipids Health Dis. 2015 Sep 13;14:105. doi: 10.1186/s12944-015-0111-5 (PMC4568070; doi:10.1186/s12944-015-0111-5)

**Additional file**

**Table S1. The list of selected SNPs**

| Gene | SNP | Chr | Position | MAF | HWE  *P*-value | Reference No. |
| --- | --- | --- | --- | --- | --- | --- |
| *APOA5* | rs2266788 | 11 | 116660686 | 0.216 | 0.9396 | 18 |
| *APOA5* | rs662799 | 11 | 116663707 | 0.293 | 0.2399 | 9 |
| *APOE* | rs769450 | 19 | 45410444 | 0.202 | 0.2632 | 24 |
| *APOE* | rs7412 | 19 | 45412079 | 0.057 | 1 | 9 |
| *CETP* | rs1800775 | 16 | 56995236 | 0.453 | 1 | 33 |
| *CETP* | rs708272 | 16 | 56996288 | 0.393 | 0.0849 | 9 |
| *GNB3* | rs5443 | 12 | 6954875 | 0.489 | 0.3030 | 9 |
| *LEPR* | rs1137101 | 1 | 66058513 | 0.145 | 0.9177 | 35 |
| *ADIPOQ* | rs1501299 | 3 | 186571123 | 0.307 | 0.5870 | 35 |
| *ADIPOQ* | rs2241766 | 3 | 186570892 | 0.297 | 0.6214 | 35 |
| *ADIPOQ* | rs266729 | 3 | 186559474 | 0.262 | 0.9469 | 35 |
| *LMNA* | rs4641 | 1 | 156107534 | 0.233 | 0.4195 | 34 |
| *ADRB2* | rs1042714 | 5 | 148206473 | 0.091 | 0.2101 | 36 |
| *ADRB3* | rs4994 | 8 | 37823798 | 0.156 | 0.6262 | 37 |
| *PPARGC1A* | rs8192678 | 4 | 23815662 | 0.434 | 0.4280 | 9 |
| *FABP2* | rs1799883 | 4 | 120241902 | 0.366 | 0.7361 | 31 |
| *ENPP1* | rs1044498 | 6 | 132172368 | 0.096 | 0.0356 | 32 |

Chr, chromosome; SNP, single nucleotide polymorphism; MAF, minor allele frequency; HWE, Hardy-Weinberg equilibrium

**Table S2. The logistic regression results for MetS and its components (Model 1^a^).**

| Gene (Chr) | SNP | Chr | WC | | TG | | HDL | | BP | | Glucose | | MetS | |
| --- | --- | --- | --- | --- | --- | --- | --- | --- | --- | --- | --- | --- | --- | --- |
|  |  |  | OR | *P*-value | OR | *P*-value | OR | *P*-value | OR | *P*-value | OR | *P*-value | OR | *P*-value |
| *APOA5* | rs2266788 | 11 | 0.954 | 0.6544 | 1.365 | 0.0045 | 1.118 | 0.4063 | 1.165 | 0.1431 | 1.101 | 0.4065 | 1.381 | 0.0069 |
| *APOA5* | rs662799 | 11 | 0.971 | 0.7550 | **1.609** | **3.25E-06** | **1.510** | **7.20E-04** | 1.033 | 0.7379 | 1.042 | 0.7019 | **1.498** | **2.90E-04** |
| *APOE* | rs769450 | 19 | 0.894 | 0.3091 | **0.674** | **0.0015** | 1.072 | 0.6235 | 0.869 | 0.2080 | 0.994 | 0.9630 | 0.759 | 0.0427 |
| *APOE* | rs7412 | 19 | 1.099 | 0.6081 | 1.234 | 0.2782 | 0.879 | 0.6129 | 0.824 | 0.3132 | 0.865 | 0.4998 | 0.898 | 0.6362 |
| *CETP* | rs1800775 | 16 | 1.016 | 0.8541 | 1.059 | 0.5310 | 1.244 | 0.0523 | 1.077 | 0.3881 | 0.987 | 0.8921 | 1.023 | 0.8262 |
| *CETP* | rs708272 | 16 | 0.989 | 0.9037 | 0.894 | 0.2227 | 0.746 | 0.0109 | 0.871 | 0.1108 | 1.022 | 0.8232 | 0.963 | 0.7135 |
| *GNB3* | rs5443 | 12 | 0.858 | 0.0748 | 0.931 | 0.4390 | 1.066 | 0.5677 | 1.059 | 0.5047 | 1.134 | 0.1880 | 0.985 | 0.8838 |
| *LEPR* | rs1137101 | 1 | 0.861 | 0.2333 | 0.882 | 0.3539 | 0.758 | 0.1120 | 1.053 | 0.6766 | 0.999 | 0.9967 | 0.822 | 0.2004 |
| *ADIPOQ* | rs1501299 | 3 | 0.904 | 0.2759 | 0.972 | 0.7723 | 1.039 | 0.7519 | 0.892 | 0.2217 | 1.220 | 0.0518 | 1.074 | 0.5109 |
| *ADIPOQ* | rs2241766 | 3 | 0.979 | 0.8158 | 0.846 | 0.0964 | 0.883 | 0.3144 | 0.973 | 0.7637 | 0.955 | 0.6601 | 0.893 | 0.3113 |
| *ADIPOQ* | rs266729 | 3 | 1.105 | 0.2976 | 1.126 | 0.2456 | 1.124 | 0.3455 | 1.139 | 0.1756 | 1.038 | 0.7309 | 1.200 | 0.1037 |
| *LMNA* | rs4641 | 1 | 1.054 | 0.6201 | 1.032 | 0.7802 | 0.951 | 0.7206 | 0.981 | 0.8585 | 1.310 | 0.0188 | 1.073 | 0.5716 |
| *ADRB2* | rs1042714 | 5 | 1.364 | 0.0434 | 0.833 | 0.2869 | 0.698 | 0.1093 | 1.229 | 0.1853 | 1.516 | 0.0119 | 1.107 | 0.5749 |
| *ADRB3* | rs4994 | 8 | 1.242 | 0.0652 | 1.096 | 0.4667 | 0.974 | 0.8685 | 1.235 | 0.0739 | 1.146 | 0.2970 | 1.268 | 0.0799 |
| *PPARGC1A* | rs8192678 | 4 | 1.147 | 0.1214 | 1.025 | 0.7940 | 1.122 | 0.3169 | 0.938 | 0.4724 | 1.100 | 0.3368 | 1.099 | 0.3699 |
| *FABP2* | rs1799883 | 4 | 0.907 | 0.2842 | 1.099 | 0.3311 | 1.001 | 0.9959 | 0.791 | 0.0112 | 0.854 | 0.1257 | 0.966 | 0.7498 |
| *ENPP1* | rs1044498 | 6 | 1.159 | 0.2873 | 1.091 | 0.5525 | 1.449 | 0.0248 | 1.012 | 0.9327 | 1.015 | 0.9239 | 1.261 | 0.1447 |

MetS components cut-off: Waist >90 Cm for men, TG > 150 mg/dL, HDL < 40 mg/dL, BP: SBP > 130 mmHg or DBP > 85 mmHg, Glucose > 100 mg/dL

^a^Model 1: Adjusted for age and region of recruitment. WC: waist circumference, TG: Triglyceride, HDL: High density lipoprotein, BP: Blood pressure, MetS: Metabolic syndrome.

Bold indicates significance of Bonfferoni adjusted *P*-value (< 0.0029)

**Table S3. The logistic regression results for MetS and its components (Model 2^a^).**

| Gene (Chr) | SNP | Chr | WC | | TG | | HDL | | BP | | Glucose | | MetS | |
| --- | --- | --- | --- | --- | --- | --- | --- | --- | --- | --- | --- | --- | --- | --- |
|  |  |  | OR | *P*-value | OR | *P*-value | OR | *P*-value | OR | *P*-value | OR | *P*-value | OR | *P*-value |
| *APOA5* | rs2266788 | 11 | 0.978 | 0.8394 | **1.402** | **0.0027** | 1.22 | 0.1488 | 1.154 | 0.1826 | 1.161 | 0.2119 | 1.427 | 0.0036 |
| *APOA5* | rs662799 | 11 | 1.009 | 0.9247 | **1.726** | **2.65E-07** | **1.654** | **7.04E-05** | 1.016 | 0.8766 | 1.100 | 0.3927 | **1.603** | **3.96E-05** |
| *APOE* | rs769450 | 19 | 0.893 | 0.3130 | **0.653** | **7.72E-04** | 1.016 | 0.9152 | 0.896 | 0.3316 | 0.979 | 0.8714 | 0.757 | 0.0448 |
| *APOE* | rs7412 | 19 | 1.096 | 0.6252 | 1.294 | 0.1929 | 0.9377 | 0.8030 | 0.793 | 0.2402 | 0.879 | 0.5609 | 0.943 | 0.7996 |
| *CETP* | rs1800775 | 16 | 1.023 | 0.7928 | 1.041 | 0.6691 | 1.279 | 0.0358 | 1.094 | 0.3075 | 0.978 | 0.8199 | 1.018 | 0.8660 |
| *CETP* | rs708272 | 16 | 0.994 | 0.9434 | 0.908 | 0.3052 | 0.7085 | 0.0044 | 0.867 | 0.1064 | 1.049 | 0.6242 | 0.969 | 0.7623 |
| *GNB3* | rs5443 | 12 | 0.857 | 0.0781 | 0.934 | 0.4683 | 1.057 | 0.6366 | 1.063 | 0.4860 | 1.147 | 0.1663 | 0.973 | 0.7962 |
| *LEPR* | rs1137101 | 1 | 0.873 | 0.2874 | 0.880 | 0.3571 | 0.7438 | 0.1015 | 1.027 | 0.8336 | 0.986 | 0.9190 | 0.805 | 0.1692 |
| *ADIPOQ* | rs1501299 | 3 | 0.899 | 0.2602 | 0.953 | 0.6391 | 1.048 | 0.7062 | 0.899 | 0.2723 | 1.225 | 0.0553 | 1.082 | 0.4782 |
| *ADIPOQ* | rs2241766 | 3 | 1.008 | 0.9351 | 0.871 | 0.1813 | 0.8677 | 0.2684 | 1.001 | 0.9905 | 0.960 | 0.7062 | 0.913 | 0.4236 |
| *ADIPOQ* | rs266729 | 3 | 1.075 | 0.4597 | 1.081 | 0.4581 | 1.102 | 0.4494 | 1.129 | 0.2163 | 1.037 | 0.7410 | 1.162 | 0.1876 |
| *LMNA* | rs4641 | 1 | 1.085 | 0.4471 | 1.061 | 0.6076 | 0.9716 | 0.8437 | 0.984 | 0.8836 | 1.275 | 0.0410 | 1.108 | 0.4189 |
| *ADRB2* | rs1042714 | 5 | 1.378 | 0.0396 | 0.854 | 0.3640 | 0.7889 | 0.2942 | 1.161 | 0.3447 | 1.492 | 0.0184 | 1.139 | 0.4777 |
| *ADRB3* | rs4994 | 8 | 1.270 | 0.0472 | 1.103 | 0.4517 | 0.9787 | 0.8946 | 1.234 | 0.0831 | 1.134 | 0.3567 | 1.281 | 0.0765 |
| *PPARGC1A* | rs8192678 | 4 | 1.136 | 0.1565 | 1.011 | 0.9091 | 1.14 | 0.2716 | 0.922 | 0.3712 | 1.079 | 0.4543 | 1.076 | 0.4949 |
| *FABP2* | rs1799883 | 4 | 0.932 | 0.4461 | 1.122 | 0.2472 | 1.035 | 0.7788 | 0.799 | 0.0177 | 0.833 | 0.0869 | 0.989 | 0.9186 |
| *ENPP1* | rs1044498 | 6 | 1.146 | 0.3422 | 1.131 | 0.4244 | 1.407 | 0.05228 | 1.031 | 0.8335 | 1.033 | 0.8419 | 1.278 | 0.1366 |

MetS components cut-off: Waist >90 Cm for men, TG > 150 mg/dL, HDL < 40 mg/dL, BP: SBP > 130 mmHg or DBP > 85 mmHg, Glucose > 100 mg/dL

^a^Model 2: Adjusted for age, region of recruitment, smoking, alcohol and physical activity.

WC: waist circumference, TG: Triglyceride HDL: High density lipoprotein, BP: Blood pressure, MetS: Metabolic syndrome.

Bold indicates significance of Bonfferoni adjusted *P*-value (< 0.0029)

**Table S4. The linear regression results of significant SNPs for quantitative trait of MetS components.**

| Gene | SNP | Chr | Model | WC | | | TG | | HDL | | SBP | | DBP | | Glucose | |
| --- | --- | --- | --- | --- | --- | --- | --- | --- | --- | --- | --- | --- | --- | --- | --- | --- |
|  |  |  |  | BETA | *P*-value | BETA | | *P*-value | BETA | *P*-value | BETA | *P*-value | BETA | *P*-value | BETA | *P*-value |
| *APOA5* | rs662799 | 11 | Model 1^a^ | -0.141 | 0.6882 | **19.550** | | **1.27E-06** | **-1.952** | **4.22E-04** | 0.633 | 0.3097 | 0.237 | 0.6219 | 0.428 | 0.5795 |
|  |  |  | Model 2^b^ | 0.205 | 0.6725 | **21.550** | | **8.39E-08** | **-2.031** | **2.35E-04** | 0.534 | 0.3965 | 0.205 | 0.6725 | 0.701 | 0.3659 |
| *APOE* | rs769450 | 19 | Model 1^a^ | -0.279 | 0.4849 | -12.660 | | 0.0060 | 0.471 | 0.4542 | -0.856 | 0.2272 | -0.506 | 0.3556 | 1.315 | 0.1331 |
|  |  |  | Model 2^b^ | -0.425 | 0.4383 | -13.030 | | 0.0043 | 0.729 | 0.2427 | -0.707 | 0.3205 | -0.425 | 0.4383 | 1.013 | 0.2459 |

WC: waist circumference, TG: Triglyceride HDL: High density lipoprotein, SBP: Systolic Blood pressure, DBP: Diastolic blood pressure

^a^Model 1: Adjusted for age and region of recruitment. ^b^Model 2: Further adjusted for smoking, alcohol and physical activity.

Bold indicates significance of Bonfferoni adjusted *P*-value (< 0.0029)

**Figure S1. TG level difference between health-related behaviors.** The comparison of TG level difference between the stratified smoking, drinking and physical activity in (A) rs662799 genotype and (B) rs769450 genotype. Asterisk indicates the difference of TG level in each categorized health-related behaviors according to the significance (*t-*test, **P*-value < 0.05, ***P*-value < 0.01, ****P*-value <0.001); All error bars represent standard error. Categorized drinking stage cut-offs, alcohol(g) / week: Non drinker = 0, light drinker < 98g, moderate drinker < 196g, heavy drinker was not shown. PA: Physical Activity


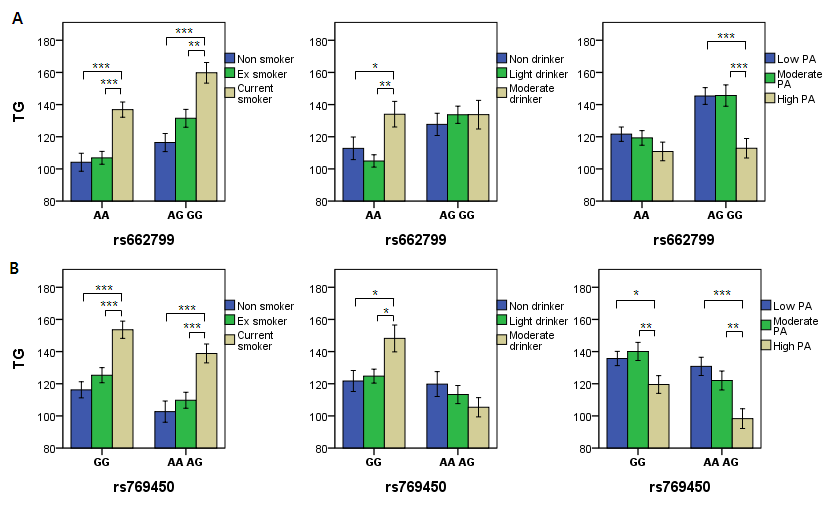

Supplement: Additional file 1: Table S1. — The list of selected SNPs. Table S2. The logistic regression results for MetS and its components (Model 1). Table S3. The logistic regression results for MetS and its components (Model 2). Table S4. The linear regression results of significant SNPs for quantitative trait of MetS components. Figure S1. TG level difference between health-related behaviors. (DOCX 75 kb) [file 12944_2015_111_MOESM1_ESM.docx]
